# Seroprevalence of SARS-CoV-2-specific anti-spike IgM, IgG, and anti-nucleocapsid IgG antibodies during the second wave of the pandemic: A population-based cross-sectional survey across Kashmir, India

**DOI:** 10.3389/fpubh.2022.967447

**Published:** 2022-10-06

**Authors:** Kouser Sideeq Lone, S. Muhammad Salim Khan, Mariya Amin Qurieshi, Sabhiya Majid, Mohammad Iqbal Pandit, Inaamul Haq, Javid Ahmad, Ashfaq Ahmad Bhat, Khalid Bashir, Sufoora Bilquees, Anjum Bashir Fazili, Muzamil Hassan, Yasmeen Jan, Rauf-ur Rashid Kaul, Zahid Ali Khan, Beenish Mushtaq, Fouzia Nazir, Uruj Altaf Qureshi, Malik Waseem Raja, Mahbooba Rasool, Anjum Asma, Arif Akbar Bhat, Iqra Nisar Chowdri, Shaista Ismail, Asif Jeelani, Misbah Ferooz Kawoosa, Mehvish Afzal Khan, Mosin Saleem Khan, Rafiya Kousar, Ab Aziz Lone, Shahroz Nabi, Tanzeela Bashir Qazi, Rouf Hussain Rather, Iram Sabah, Ishtiyaq Ahmad Sumji

**Affiliations:** ^1^Department of Community Medicine, Government Medical College Srinagar, Srinagar, India; ^2^Department of Biochemistry, Government Medical College Srinagar, Srinagar, India; ^3^Department of Community Medicine, Sher-i-Kashmir Institute of Medical Sciences, Srinagar, India; ^4^Department of Community Medicine, SKIMS Medical College Srinagar, Srinagar, India; ^5^Department of Community Medicine, Government Medical College Baramulla, Baramulla, India; ^6^Department of Community Medicine, Government Medical College Anantnag, Anantnag, India; ^7^Directorate of Health Services Kashmir, Government of Jammu and Kashmir, Srinagar, India

**Keywords:** SARS-CoV-2, COVID-19, antibodies, seroprevalence, epidemiology

## Abstract

**Background:**

Within Kashmir, which is one of the topographically distinct areas in the Himalayan belt of India, a total of 2,236 cumulative deaths occurred by the end of the second wave. We aimed to conduct this population-based study in the age group of 7 years and above to estimate the seropositivity and its attributes in Kashmir valley.

**Methods:**

We conducted a community-based household-level cross-sectional study, with a multistage, population-stratified, probability-proportionate-to-size, cluster sampling method to select 400 participants from each of the 10 districts of Kashmir. We also selected a quota of healthcare workers, police personnel, and antenatal women from each of the districts. Households were selected from each cluster and all family members with age 7 years or more were invited to participate. Information was collected through a standardized questionnaire and entered into Epicollect 5 software. Trained healthcare personnel were assigned for collecting venous blood samples from each of the participants which were transferred and processed for immunological testing. Testing was done for the presence of SARS-CoV-2-specific anti-spike IgM, IgG antibodies, and anti-nucleocapsid IgG antibodies. Weighted seropositivity was estimated along with the adjustment done for the sensitivity and specificity of the test used.

**Findings:**

The data were collected from a total of 4,229 participants from the general population within the 10 districts of Kashmir. Our results showed that 84.84% (95% CI 84.51–85.18%) of the participants were seropositive in the weighted imputed data among the general population. In multiple logistic regression, the variables significantly affecting the seroprevalence were the age group 45–59 years (odds ratio of 0.73; 95% CI 0.67–0.78), self-reported history of comorbidity (odds ratio of 1.47; 95% CI 1.33–1.61), and positive vaccination history (odds ratio of 0.85; 95% CI 0.79–0.90) for anti-nucleocapsid IgG antibodies. The entire assessed variables showed a significant role during multiple logistic regression analysis for affecting IgM anti-spike antibodies with an odds ratio of 1.45 (95% CI 1.32–1.57) for age more than 60 years, 1.21 (95% CI 1.15–1.27) for the female gender, 0.87 (95% CI 0.82–0.92) for urban residents, 0.86 (95% CI 0.76–0.92) for self-reported comorbidity, and an odds ratio of 1.16 (95% CI 1.08–1.24) for a positive history of vaccination. The estimated infection fatality ratio was 0.033% (95% CI: 0.034–0.032%) between 22 May and 31 July 2021 against the seropositivity for IgM antibodies.

**Interpretation:**

During the second wave of the SARS-CoV-2 pandemic, 84.84% (95% CI 84.51–85.18%) of participants from this population-based cross-sectional sample were seropositive against SARS-CoV-2. Despite a comparatively lower number of cases reported and lower vaccination coverage in the region, our study found such high seropositivity across all age groups, which indicates the higher number of subclinical and less severe unnoticed caseload in the community.

## Introduction

The second wave of the COVID-19 pandemic caused by the SARS-CoV-2 started in India in the middle of March 2021 (1, 2). In India, the maximum number of deaths, that is about 50% of total reported COVID-19 deaths, happened during the peak of this second wave between April and June 2021 (1). The compiled data for the total number of deaths by the Ministry of Health and Family Welfare shows that 235,986 persons died of COVID-19 from April to June in 2021 (1). Worldwide, India has the third highest number of deaths and highest number of cases after the United States since the inception of COVID-19 disease. The total death toll for India was 448,846 till October 2021 (3). The death toll peaked in May 2021 when 120,770 persons lost their lives to COVID-19 in just 1 month (1). In Jammu and Kashmir, the northernmost part of India, a total of 4,398 deaths and 323,499 confirmed cases of COVID-19 were reported by the end of the second wave since the beginning of the outbreak, with more than 60% of cases and around 50% of deaths occurring only from Kashmir division (1, 4). In October-November 2020, the first pan-Kashmir serosurveillance study found a seroprevalence of 36.7% among the general population aged more than 18 years (5). Since the start of the pandemic, the lockdown was imposed in Kashmir with periodic systematic relaxations. Similar restrictions were imposed during the second wave of the pandemic, in April-May 2021. A few months before the start of the second wave, COVID-19 vaccines were introduced for use in healthcare personnel. By March 2021, every adult >18 years of age was eligible to receive the vaccine. As per official statistics, around 20% of the population were vaccinated with two doses by July 2021 (6, 7).

We aimed to do a pan-Kashmir household serosurvey designed to understand the status of SARS-CoV-2 infection in the general population during this complex period of the second wave of the pandemic. The objectives of this second serosurvey were to estimate and understand the change among the general Kashmiri population regarding the seroprevalence of SARS-CoV-2 antibodies. In the study, we also included the school-age groups with ages more than or equal to 7 years as the inclusion criteria for the study, as this age group has not been studied in previous research regarding the seroprevalence of SARS-CoV-2. In addition, we also extended the study to include a quota of frontline workers and pregnant women from each district.

## Methods

### Study settings

**Figure 1:**
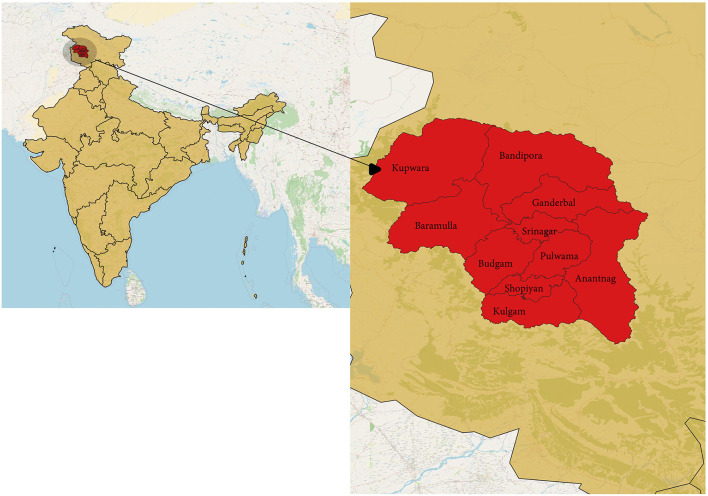
The study area in the context of the global map.

**Figure 2:**
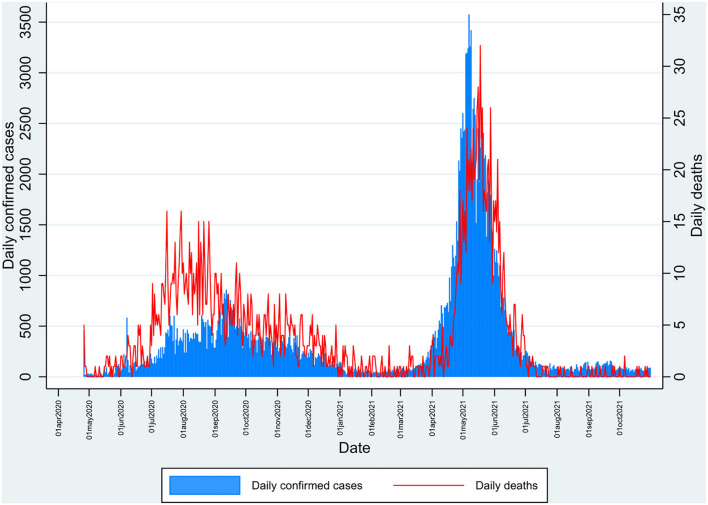
Daily number of reported cases and deaths in Kashmir during the pandemic of SARS-CoV-2 from April 2020 to October 2021 depicting the first and second peak waves.

The study was done in Kashmir province, a part of the union territory of Jammu and Kashmir, India. Kashmir is the northernmost part of the country, situated in the Himalayan belt on the global map. The region is divided into two major provinces: Jammu province and Kashmir province. The territory has approximately a population of 12.3 million, with Kashmir province being the most populous among the two divisions with about 7 million people; the Jammu province has a total population of around 5.3 million (8). We chose the Kashmir division for our study, which has 10 districts (Figure 1). India had its second wave of COVID-19 pandemic infection in March-August 2021, which led to the maximum number of deaths since the outbreak. The same scenario was seen in Jammu and Kashmir, with the major number of deaths occurring in Kashmir (Figure 2) (1).

### Study design

We designed a population-based cross-sectional study. We covered all 10 districts of Kashmir valley (Figure 1). In all 10 districts, the study was primarily focused on the general population. We extended the study to include a quota of frontline workers and antenatal women from each district to compare the results of these exposed groups. The antenatal women were the strata in which the Government did not approve vaccines against COVID-19 until July 2021. Our study began in the same month. Pregnant females being the least exposed to vaccination among adults, we added a quota of 50 pregnant females within each district to assess the seroprevalence within this vulnerable group. Among the frontline workers, we included healthcare workers and police personnel from each district. Data collection was completed in 11 days, from 5 July 2021 to 15 July 2021.

### Participants

#### General population

We used a multistage, stratified, cluster random sampling method to enroll participants. We stratified clusters within all 10 districts into urban and rural strata. From each district, 10 clusters were selected using the probability-proportionate-to-size sampling method. A total of 100 clusters were thus selected. Each cluster was divided into four equal areas, and within each of these areas, a central location was chosen as the starting point, and the first household was selected randomly by spinning the bottle method in each area. After that, we approached consecutive households to enroll at least 10 eligible participants in each area to obtain at least 40 participants per cluster. All individuals ≥7 years of age were eligible to participate in the study. We invited all eligible candidates in a household to participate.

#### Frontline workers

In each district, 100 healthcare workers and 100 police personnel were selected. The healthcare workers were selected from district-level health facilities within each district with consecutive sampling until 100 participants were enrolled. Police personnel was also selected from the district police lines with consecutive sampling until 100 participants were enrolled within each district.

#### Pregnant females

In each district, 50 pregnant females were consecutively selected from the district-level health facility.

### Sample size

For the general population, we calculated the minimum sample size based on an anticipated seroprevalence of 50% (a nationwide survey had reported a seroprevalence of 24.1% for January 2021) (9). We used an absolute precision of 2.5% and a design effect of 2. OpenEpi (www.openepi.com) was used to calculate the sample size, and using the above parameters, the sample required was 3,069 participants (10). After adjusting for a possible non-response of 10%, a sample of 3,376 was calculated. For feasibility, we aimed for a 4,000 total sample size. Finally, 4,229 participants were enrolled from the total of 10 districts of Kashmir valley, and every family member with age ≥7 years in each selected household was included.

### Variables

The primary outcome variables of interest were SARS-CoV-2 specific antibodies which included anti-spike IgG, IgM, and anti-nucleocapsid IgG antibodies. We obtained information on demographic and other variables from participants that included their age, gender, history of comorbidity, history of COVID-19 testing with RTCPR, and vaccination history against SARS-CoV-2 with any of the approved vaccines in India.

### Procedure

The eligible candidates were informed about the purpose and the procedure of the study. Written informed consent was taken from each of the study participants for voluntary participation. Specifically, trained health personnel was involved in data collection to interview all participants. For participants with an age <18 years, consent was taken from the guardian. The interview responses were recorded in an Epicollect 5 form (11). After the interview was complete, 3 to 5 ml of venous blood was collected by a trained phlebotomist from the antecubital vein of the participant under complete aseptic precautions. The collected blood was immediately transferred into a collection tube containing a clot activator which was then left standing, undisturbed, for at least 30 min for clot formation. After this, the sample was transported under strict cold chain protocol to the public health lab in the Department of Community Medicine GMC Srinagar on the same day for centrifugation. In the public health laboratory, the samples of serum after separation were stored under a cold chain with a temperature maintained between 2 and 8 degrees Celsius. For further processing and analysis, the Centrifuged serum samples were transported to a central laboratory. Serum samples were tested for the presence of SARS-CoV-2-specific IgG anti-spike and anti-nucleocapsid antibodies and also anti-spike IgM antibodies using the Abbott SARS-CoV-2 assay. The assay uses chemiluminescent microparticle immunoassay (CMIA) to detect antibodies against the SARS-CoV-2 (12). The reported sensitivity of the assay for IgG anti-spike antibody, IgG anti-nucleocapsid, and IgM anti-spike antibodies are 99.37% (95% CI 96.50 to 99.97%), 100% (95% CI 95.89 to 100.00%), and 96·67% (90.65 to 98.86%), respectively. The specificity of the assay is 99.60% (95% CI 98.98 to 99.89%) for anti-nucleocapsid IgG, 99.55% (95% CI 99.15 to 99.76%) for anti-spike IgG, and 99.56% (95% CI 99.25 to 99.74%) for IgM antibody (12).

### Antibody cut-off levels for labeling seropositivity

CMIA we used included the quantitative measurement of IgG antibodies against the spike receptor-binding domain of SARS-CoV-2 and qualitative measurement for anti-nucleocapsid IgG and anti-spike IgM antibodies. The result for antibodies was labeled according to the cut-off recommended by the manufacturer; assay results equal to or more than the index value of 1.4 for anti-nucleocapsid SARS-CoV-2-specific IgG antibody was labeled as positive, for anti-spike IgG antibody, results equal to or more than 50 Au/ml was labeled as positive, for anti-spike IgM assay, results equal to or above the cut-off index value of 1 was labeled as positive (12).

### Statistical methods

The available case, and complete case analysis was done, and the multiple data imputations were done with the help of computer software to compensate for the missing information in the data, after which the data were again analyzed. We calculated both unweighted (adjusted for clustering) and weighted seroprevalence estimates, and both were reported in percentages. The Agresti–Coull procedure was used to calculate a 95% CI for seroprevalence estimates (13). For weighted estimates, survey weights (inverse of sampling probability) were calculated for which the estimated population of the districts using the census 2011 data and growth rates from the Sample Registration System were used. For these Survey weights, post-stratification weights were calculated by adjusting the age and sex structures and non-response. Further adjustment for the weighted seroprevalence estimates was made to calculate weighted seroprevalence adjusted for test performance. This was done using the formula: Weighted seroprevalence adjusted for test performance= (Weighted seroprevalence + Test specificity-1)/(Test sensitivity + Test specificity-1). We used the manufacturer-provided sensitivity and specificity in the above formula. The extremes of the manufacturer provided 95% CI of the test sensitivity and specificity (upper limit of sensitivity, lower limit of specificity; and lower limit of sensitivity, upper limit of specificity) was used to report sensitivity analyses. Univariate logistic regression analysis was used to calculate the odds ratio for estimating significant attributes of seropositivity in the general population. Further, we did the adjusted analysis by multiple logistic regression with the variables to estimate the strength and significance of the association.

The data were analyzed using Stata V.15 (StataCorp. 2017. Stata Statistical Software: Release 15. College Station, Texas: StataCorp) (14).

The estimated number of SARS-CoV-2 infections was calculated by multiplying the estimated population of Kashmir with the weighted seroprevalence from the imputed data adjusted for test performance. The discrete seroprevalence and also simultaneous seropositivity for all three antibodies (anti-nucleocapsid IgG, anti-spike IgG antibody, and anti-spike IgM antibody) were used to estimate the number of infections. For estimation of the number of infections per reported case, we divided the estimated number of SARS-CoV-2 infections by the reported cumulative number of COVID-19 cases at 2 weeks (26 June 2021) and at 5 days before (5 July 2021) the survey date for calculations using the seropositivity rate of anti-spike IgG antibody (5 days for the minimum time and 2 weeks for the maximum time for appearance of antibody; before the median time of the sample collection). For estimating the number of infections per reported case using the seropositivity of anti-nucleocapsid IgG antibody, the denominator used was the reported number of cases in the previous 6 months from 23 December 2020 to 26 June 2021 and from 23 December 2020 to 5 July 2021; while using the seropositivity for IgM, the denominator used was the cumulative cases in the previous 8 weeks (from 1 May 2021 till 26 June 2021 and from 1 May 2021 till 5 July 2021). The infection fatality rate was calculated by dividing the reported number of deaths by the number of estimated infections while assuming a 3-week lag time from infection to death. The cumulative number of deaths reported till 31 July 2021 was used for calculation with seropositivity for anti-spike IgG antibody. For calculations with anti-nucleocapsid IgG and anti-spike IgM antibodies, we used the number of deaths between 13 January and 31 July and the cumulative deaths between 22 May and 31 July, respectively.

## Results

**Figure 3:**
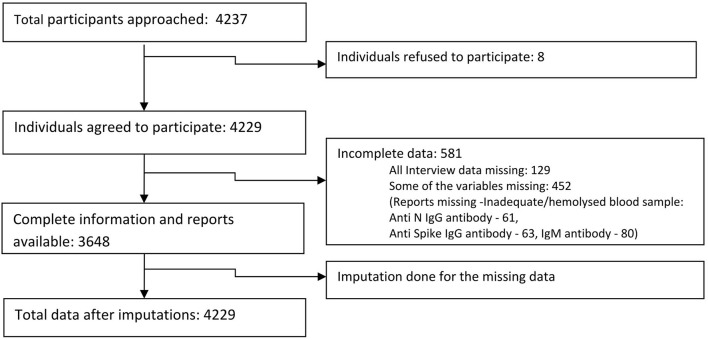
Participant flow chart.

We approached 4,237 participants in the general population from the 10 districts of Kashmir valley, out of which 4,229 (99.8%) agreed to participate. We obtained complete information from 3,648/4,229 (86.3%) participants (Figure 3). Among the study participants, 1,638 (39.95%) were 18 to 44 years old and 1,087 (26.51%) were 7 to 17 years old in the available case analysis (Table 1). The number of males and females was approximately equal, and 3,161 (74·75%) of the participants lived in rural areas. A total of 171 (10.62%) participants out of 1,612 (39.45%) who had tested for COVID-19 reported a positive COVID-19 test. A total of 2,225 (55.78%) participants reported a history of vaccination with at least one dose of approved COVID-19 vaccines. Among the participants in the available case analysis, the seropositivity for any of the three antibodies (anti-nucleocapsid IgG/anti-spike IgG /anti-spike IgM antibodies) was 85.15% (3550/4169) (Table 1, Supplementary Tables 1, 2). The majority of the participants (96.9%) gave a history of vaccination with the Covishield vaccine (ChAdOx1 nCoV-19 Corona Virus Vaccine; Recombinant). Among 1,653 unvaccinated participants, 247 did not get vaccinated because of fear of side effects (Table 1).

**Table 1:** Baseline characteristics of the study participants.

**Characteristics**	**Available case analysis**	**Complete case analysis**
	***N* (Percent)**	**(Total 3,648) *N* (Percent)**
**Age in years**	**Total 4,100**	
7–17	1,087 (26.51)	953 (26.12)
18–44	1,638 (39.95)	1,460 (40.02)
45–59	831 (20.27)	757 (20.75)
≥60	544 (13.27)	478 (13.1)
**Gender**	**Total 4,100**	
Male	2,117 (51.63)	1,886 (51.7)
Female	1,983 (48.37)	1,762 (48.3)
**Residence**	**Total 4,229**	
Urban	1,068 (25.25)	1,001 (27.44)
Rural	3,161 (74.75)	2,647 (72.56)
**Self reported history of chronic disease**	**Total 3,807**	
No	3,403 (89.39)	3,263 (89.45)
Yes	404 (10.61)	385 (10.55)
**Ever tested for COVID 19 with RTPCR**	**Total 4,086**	
No	2,474 (60.55)	2,176 (59.65)
Yes	1,612 (39.45)	1,472 (40.35)
**Results of the test**	**Total 1,610**	
Positive	171 (10.62)	153 (10.39)
Negative	1,438 (89.32)	1,318 (89.54)
Result awaited	1 (0.06)	1 (0.07)
**Symptoms in positive participants**	**Total 164**	**Total 153**
Asymptomatic	40 (24.39)	37 (24.18)
Symptomatic but treated at home without oxygen	103 (62.8)	95 (62.09)
Home treatment with oxygen	6 (3.66)	6 (3.92)
Hospitalized	15 (9.15)	15 (9.8)
**Any other family member being ever positive**	**Total 3,758**	
No	3,490 (92.87)	3,397 (93.12)
Yes	268 (7.13)	251 (6.88)
**Whether Vaccinated against COVID 19 (at least one dose)**	**Total 3,989**	
No	1,764 (44.22)	1,618 (44.35)
Yes	2,225 (55.78)	2,030 (55.65)
**Vaccine type**	**Total 2,221**	**Total 2,030**
Covaxin	50 (2.25)	45 (2.22)
Covishield	2,152 (96.89)	1,966 (96.84)
Don't know	19 (0.86)	19 (0.94)
**Reason if not vaccinated**	**Total 1,653**	**Total 1,618**
Do not believe in vaccines	21 (1.27)	20 (1.24)
Fear of side effects	247 (14.94)	246 (15.2)
Fear of side effects, Do not believe in vaccines	1 (0.06)	1 (0.062)
No response	202 (12.22)	195 (12.05)
No response, Not interested	1 (0.06)	1 (0.06)
Not applicable (<18 years or pregnant)	854 (51.664)	833 (51.48)
Not interested	145 (8.77)	144 (8.9)
Waiting for due date	181 (10.95)	177 (10.94)
Waiting for due date, Fear of side effects	1 (0.06)	1 (0.06)
**Antibody results**	**N (Percent)**	**N (Percent)**
**Anti-Nucleocapsid IgG antibodies**	**Total 4,168**	**Total 3,648**
Positive	1,142 (27.4)	1,000 (27.41)
**Anti-spike IgG antibodies**	**Total 4,166**	**Total 3,648**
Positive	3,519 (84.47)	3,120 (85.53)
**Anti-spike IgM antibodies**	**Total 4,149**	**Total 3,648**
Positive	845 (20.37)	729 (19.98)
**Any of the three antibodies**	**Total 4,169**	**Total 3,648**
Positive	3,550 (85.15)	3,144 (86.18)
**All of the three antibodies**	**Total 4,169**	**Total 3,648**
Yes	377 (9.04)	314 (8.61)

The highest seroprevalence was found for anti-spike IgG antibodies across all the categories of participant characteristics in the complete data as well as imputed data (Tables 2, 3). The seroprevalence was highest in the age group of ≥60 years for all categories of antibody except for the anti-nucleocapsid IgG antibody, which was highest in the age group of 7 to 17 years both in complete case analysis as well as imputed data analysis (Tables 2, 3). The distribution of seroprevalence was higher among females in the weighted adjusted for test performance analysis for all types of antibody positivity. Seroprevalence was higher in urban residents (Tables 2, 3). The participants with any self-reported comorbidity and those with positive RT-PCR among the tested ones had higher seroprevalence in all three antibody analyses (Table 2). Participants who were not vaccinated had a higher seroprevalence of anti-nucleocapsid IgG antibodies as compared to the vaccinated ones.

**Table 2:** Seroprevalence (unweighted and weighted) of the complete cases by selected characteristics.

**Variables**	**Seropositivity for**		**Seropositivity for**		**Seropositivity for**	
	**Anti– N IgG antibody**		**Anti-spike IgG antibodies**		**Anti-spike IgM antibodies**	
	**Unweighted percentage (95% CI)^∧^**	**Weighted percentage (95% CI)**	**Unweighted percentage (95% CI)^∧^**	**Weighted percentage (95% CI)**	**Unweighted percentage (95% CI)^∧^**	**Weighted percentage (95% CI)**
**Age groups (years)**
7– 17	30.43 (26.53– 34.64)	31.19 (30.41–31.98)	80.17 (76.25–83.58)	81.13 (80.5–881.66)	17 (14.52–19.80)	18.74 (18.23–19.26)
18–44	27.4 (24.53–30.47)	27.33 (26.70–27.97)	84.32 (82.35–86.10)	84.4 (84.04–84.76)	18.63 (16.38– 21.12)	18.32 (17.86–18.78)
45–59	22.46 (19.71–25.46)	23.2 (22.57–23.84)	91.02 (88.63–92.94)	90.22 (89.79–90.64)	22.99 (19.95–26.33)	23.35 (22.83–23.88)
≥60	29.29 (24.82–34.19)	29.28 (28.48–30.10)	91.21 (87.42–93.94)	92.41 (91.95–92.84)	25.31 (21.09–30.06)	27.65 (26.90–28.42)
**Gender**
Male	27.31 (24.53–30.27)	27.97 (27.36–28.59)	84.89 (82.47–87.03)	83.62 (83.20–84.02)	19.51 (17.47–21.73)	18.4 (17.92–18.88)
Female	27.53 (24.70–30.55)	28.28 (27.76–28.80)	86.21 (83.96–88.19)	86.21 (85.86–86.55)	20.49 (18.66–22.44)	21.51 (21.11–21.91)
**Residence**
Urban	29.87 (25.77–34.31)	30.34 (29.07–31.65)	87.65 (87.06–88.22)	87.65 (87.06–88.22)	21.37 (18.37–24.72)	21.66 (21.00–
Rural	26.48 (23.71–29.44)	27.01 (26.63–27.39)	83.46 (83.11–83.80)	83.46 (83.11–83.80)	19.45 (17.874–21.14)	18.99 (18.71–19.27)
**Comorbidity**
No	26.87 (24.56–29.32)	27.6 (27.16–28.04)	83.97 (83.66–84.28)	83.97 (83.66–84.28)	19.73 (18.16–21.40)	19.74 (19.42–20.06)
Yes	31.94 (26.67–37.72)	33.18 (31.63–34.76)	93.4 (92.87–93.90)	93.4 (92.87–93.90)	22.07 (18.95–25.55)	21.19 (20.61–21.78)
**RT PCR Status**
Negative	25.42 (22.09–29.05)	27.1 (26.23–27.99)	87.32 (86.80–87.82)	87.32 (86.80–87.82)	20.4 (17.91–23.14)	20.61 (20.05–21.19)
Positive	50.32 (41.20–59.42)	49.34 (47.38–51.31)	92.75 (91.68–93.70)	92.75 (91.68–93.70)	28.1 (21.43–35.90)	22.53 (20.96–24.18)
**Vaccinated with at least one done**						
No	29.97 (26.63–33.54)	30.13 (29.41–30.87)	80.74 (80.26–81.21)	80.74 (80.26–81.21)	17.73 (15.72–19.95)	18.3 (17.95–18.66)
Yes	25.36 (23.14–27.72)	26.04 (25.61–26.47)	89.08 (88.79–89.35)	89.08 (88.79–89.35)	21.7 (19.68–24.01)	21.49 (21.03–21.97)

^∧^ Adjusted for clustering.

**Table 3:** Seroprevalence in the imputed data (unweighted, weighted, and adjusted for the sensitivity and specificity of the test used).

**Variables**	**Unweighted Seroprevalence% with MI (95% CI)^∧^**	**Weighted Seroprevalence% with MI (95% CI)**	**Weighted Seroprevalence% Adjusted with lower test sensitivity and higher specificity (95%CI)**	**Weighted Seroprevalence% Adjusted with lower test specificity and higher sensitivity (95%CI)**	**Weighted Seroprevalence% Adjusted for average test sensitivity and specificity (95%CI)**
**Anti-Nucleocapsid IgG antibodies**					
**Age groups (in years)**					
7–17	29.41 (25.74–33.07)	30.34 (29.48–31.19)	30.23(29.38–31.09)	29.36(28.52–30.23)	29.96(29.11–30.81)
18–44	27.64(24.93–30.36)	27.54 (26.79–28.30)	27.44(26.69–28.19)	26.56(25.83–27.34)	27.17(26.41–27.92)
45–59	22.82(20.09–25.55)	23.36 (22.70–24.02)	23.26(22.60–23.92)	22.38(21.74–23.06)	22.99(22.33–23.65)
≥60	29.73 (25.27–34.19)	29.3 (28.0–30.30)	29.20(28.20–30.20)	28.32(27.34–29.34)	28.93(27.93–29.93)
**Gender**					
Male	27.33(24.75–29.9)	27.77 (27.16–28.37)	27.67(27.06–28.27)	26.79(26.21–27.41)	27.39(26.79–27.99)
Female	27.51(24.78–30.24)	28.23 (27.59–28.87)	28.13(27.49–28.77)	27.25(26.63–27.91)	27.86(27.22–28.50)
**Residence**					
Urban	29.22 (25.01–33.44)	29.6 (28.28–30.91)	29.50(28.18–30.81	28.62(27.32–29.95)	29.22(27.91–30.54)
Rural	26.80(24.27–29.34)	27.24 (26.80–27.67)	26.26(26.70–27.57)	26.26(25.84–26.71)	26.86(26.43–27.29)
**Comorbidity present**					
No	26.75(24.58–28.92)	27.41 (26.91–27.91)	27.13(26.81–27.81)	26.43(25.95–26.95)	27.04(26.53–27.54)
Yes	32.6 (27.57–37.64)	33.28 (31.55–35.00)	33.17(31.45–34.90	32.30(30.59–34.04)	32.90(31.18–34.62)
**RTPCR results**					
Negative	25.18 (22.13–28.23)	26.52 (25.67–27.36)	26.42(25.57–27.26)	25.54(24.71–26.41)	26.15(25.30–26.99)
Positive	51.06 (41.82–60.29)	52.4 (49.97– 54.83)	52.30(49.87–54.73)	51.42(49.01–53.88)	52.03(49.60–54.46)
**Vaccinated with at least one dose of the approved vaccines**					
No	29.7(26.56–32.83)	29.92 (29.16–30.67)	29.81(29.06–30.57)	28.94(28.20–29.71)	29.54(28.79–30.29)
Yes	25.56 (23.41–27.71)	26 (25.48–26.52)	25.90(25.38–26.42)	25.02(24.52–25.56)	25.63(25.11–26.15)
**Anti-spike IgG antibodies**					
**Age group**					
7–17	78.51 (75.11–81.91)	79.93 (79.31–80.55)	79.68(79.06–80.31)	79.07(78.45–79.69)	79.47(78.85–80.09)
18–44	83.62 (81.78–85.47)	83.92 (83.47–84.38)	83.68(83.22–84.14)	83.06(82.61–83.52)	83.47(83.01–83.92)
45–59	90.04 (87.83–92.26)	89.53 (89.07–90.00)	89.29(88.82–89.75)	88.67(88.21–89.14)	89.08(88.61–89.54)
≥60	90.33 (87.37–93.29)	91.47 (90.86–92.09)	91.23(90.62–91.85)	90.62(90.00–91.23)	91.02(90.41–91.63)
**Gender**					
Male	83.81 (81.63–85.99)	82.73 (82.28–83.18)	82.48(82.03–82.94)	81.87(81.42–82.32)	82.27(81.82–82.72)
Female	85.12 (83.14–87.1)	85.59 (85.18–86.01)	85.35(84.94–85.77)	84.74(84.32–85.15)	85.14(84.73–85.55)
**Residence**					
Urban	86.64 (83.89–89.4)	86.63 (85.96–87.29)	86.38(85.72–87.05)	85.77(85.10–86.43)	86.17(85.51–86.84)
Rural	83.7 (81.68–85.72)	82.9 (82.54–83.26)	82.66(82.30–83.02)	82.04(81.68–82.40)	82.45(82.09–82.81)
**Vaccinated with at least one dose of the approved vaccines**					
No	78.65 (76.08–81.23)	79.71 (79.17–80.24)	79.46(78.93–80.00)	78.85(78.31–79.38)	79.25(78.72–79.78)
Yes	89.15 (87.58–90.73)	88.61 (88.23–88.98)	88.36(87.99–88.74)	87.75(87.37–88.12)	88.15(87.78–88.53
**Comorbidity present**					
No	83.35 (81.55–85.15)	83.26 (82.87–83.66)	83.02(82.63–83.42)	82.41(82.01–82.80)	82.81(82.41–83.21)
Yes	93.01 (90.24–95.78)	91.64 (90.42–92.86)	91.40(90.17–92.62)	90.78(89.56–92.00)	91.18(89.96–92.41)
**RT PCR results**					
Negative	87.4 (85.35–89.45)	86.42(85.81–87.04)	86.18(85.57–86.80)	85.57(84.95–86.18)	85.97(85.35–86.58)
Positive	92.64 (87.71–97.57)	93.04(91.54–94.53)	92.80(91.30–94.29)	92.18(90.69–93.67)	92.58(91.09–94.08)
**Anti-spike IgM antibodies**					
**Age groups**					
7–17	17.7 (15.21–20.19)	19.06 (18.45–19.67)	18.80 (18.19–19.41)	18.30(17.70–18.92)	18.61(18.01–19.23)
18–44	19.25 (16.96–21.54)	18.85 (18.23–19.47)	18.54(17.97–19.22)	18.04(17.47–18.72)	18.35(17.78–19.03)
45–59	23.26 (20.13–26.4)	23.57 (22.96–24.17)	23.24(22.70–23.91)	22.74(22.21–23.42)	23.05((22.52–23.73)
≥60	24.81 (20.73–28.89)	26.7 (25.82–27.58)	26.44(25.56–27.32)	25.94(25.07–26.82)	26.25(25.38–27.13)
**Gender**					
Male	19.99 (18–21.98)	18.85 (18.31–19.39)	18.59(18.05–19.13)	18.09(17.55–18.63)	18.40(17.86–18.94)
Female	20.81 (19.01–22.6)	21.65 (21.13–22.18)	21.39(20.87–21.91	20.89(20.37–21.42)	21.21(20.68–21.73)
**Residence**					
Urban	21.37 (18.34–24.39)	21.59 (20.81–22.37)	21.33(20.55–22.11)	20.83(20.05–21.61)	21.15(20.36–21.92)
Rural	20.05 (18.45–21.65)	19.52 (19.19–19.85)	19.26(18.93–19.59)	18.76(18.43–19.10)	19.08(18.74–19.40)
**Comorbidity**					
No	20.14 (18.59–21.68)	20.05 (19.62–20.47)	19.78(19.36–20.21)	19.29(18.87–19.71)	19.60(19.17–20.02)
Yes	22.3 (18.89–25.7)	21.41 (20.10–22.71)	21.14(19.83–22.45)	20.65(19.34–21.96)	20.96(19.65–22.26)
**RT PCR results**					
Negative	20.44 (18.09–22.79)	20.69 (20.00–21.38)	20.43(19.74–21.12)	19.93(19.24–20.62)	20.25(19.55–20.93)
Positive	30.57 (22.89–38.26)	26.39 (24.30–28.49)	26.13(24.04–28.23)	25.64(23.54–27.73)	25.95(23.85–28.04)
**Vaccinated with at least one dose of the approved vaccines**					
No	18.59 (16.43–20.75)	18.84 (18.36–19.31)	18.58(18.10–19.05)	18.08(17.61–18.56)	18.39(17.92–18.86)
Yes	21.84 (19.82–23.86)	21.56 (21.00–22.13)	21.30(20.73–21.86)	20.80(20.24–21.37)	21.12(20.55–21.68)

^∧^ Adjusted for clustering; MI, Multiple Imputation.

In the weighted analysis, the age group of ≥60 years had higher seropositivity for all three antibodies together as well as seropositivity for any one of the three antibodies as compared to other age groups (Table 4). The urban residents, the participants with comorbidity, and those with positive RT-PCR test showed higher seroprevalence for all the three antibodies together as well as any one of the antibodies as compared to their counterparts in both weighted and unweighted results in all types of analyses (Table 4). Similar results were obtained for vaccinated individuals, except for the weighted analysis showing less seropositivity than the unvaccinated in the case of all three antibodies simultaneously (Table 4). The results showed 84.84% (95% CI: 84.51 to 85.18) seropositivity for any one of the three analyzed antibodies in the weighted imputed data (Table 5).

**Table 4:** Seropositivity for any one of the antibodies (Anti-Nucleocapsid IgG/Anti-spike IgG/IgM Antibodies) and all the three antibody types simultaneously.

	**Analysis of the Complete case data**, ***N*** = **3,648**				**Analysis of Data with imputations**, ***N*** = **4,229**			
**Variables**	**Percent positive for any of**		**Percent positive for all of**		**Percent positive for any of**		**Percent positive for all of**	
	**the three antibodies**		**the three antibodies**		**the three antibodies**		**the three antibodies**	
	**Unweighted percent (95% CI)^∧^**	**Weighted percent (95% CI)**	**Unweighted percent (95% CI)^∧^**	**Weighted percent (95% CI)**	**Unweighted percent (95% CI)^∧^**	**Weighted percent (95% CI)**	**Unweighted percent (95% CI)^∧^**	**Weighted percent (95% CI)**
**Age**
7– 17	80.8 (76.91–84.16)	81.71 (18.23–19.26)	7.87 (6.05–10.17)	9.35 (8.86–9.86)	79.23 (75.84–82.63)	80.55 (79.92–81.18)	8.27 (6.31–10.22)	9.45 (8.92–9.97)
18–44	84.93 (83.04–86.65)	85.04 (17.86–18.78)	7.19 (5.74– 8.98)	7.52 (7.20–7.85)	84.3 (82.53–86.08)	84.64 (84.19–85.1)	8.05 (6.37–9.74)	8.13 (7.71–8.55)
45–59	92.21 (89.91–94.02)	91.51 (22.83–23.88)	8.98 (7.32–10.97)	9.14 (8.77–9.52)	91.35 (89.32–93.39)	91.01 (90.58–91.44)	9.35 (7.5–11.2)	9.37 (8.97–9.77)
≥60	91.21 (87.42–93.94)	92.41 (26.90–28.42)	13.81 (10.46–18.01)	14.72 (14.04–15.43)	90.55 (87.58–93.52)	91.81 (91.24–92.38)	13.5 (10.07–16.93)	14.1 (13.28–14.92)
**Gender**								
Male	85.42 (83.01–87.54)	84.2 (17.92–18.88)	8.32 (6.92–9.98)	8.08 (7.82–8.36)	84.4 (82.26–86.55)	83.41 (82.95–83.86)	8.98 (7.48–10.47)	8.53 (8.2–8.87)
Female	87 (84.85–88.89)	86.92 (21.11–21.91)	8.91 (7.55–10.48)	9.75 (9.47–10.03)	86.06 (84.16–87.96)	86.43 (86.04–86.83)	9.22 (7.79–10.65)	9.9 (9.48–10.33)
**Residence**								
Urban	88.21 (86.01–90.1)	88.08 (21.00–22.33)	10.78 (8.43–13.69)	11.09 (10.52–11.69)	87.41 (85.15–89.66)	87.35 (86.7–88)	10.44 (7.95–12.93)	10.77 (10.15–11.38)
Rural	85.42 (83.07–87.48)	84.22 (18.71–19.27)	7.782 (6.68–9.04)	7.78 (7.58–7.97)	84.46 (82.48–86.45)	83.67 (83.31–84.03)	8.64 (7.34–9.93)	8.44 (8.18–8.7)
**Co morbidity**								
No	85.13 (83.20–86.87)	84.69 (19.42–20.06)	8.243 (7.09–9.56)	8.61 (8.36–8.87)	84.17 (82.43–85.92)	84.07 (83.7–84.44)	8.71 (7.49–9.92)	8.9 (8.58–9.21)
Yes	95.06 (92.50–96.78)	93.4 (20.61–21.78)	11.68 (1.522–15.06)	11.47 (10.85–12.11)	93.28 (90.69–95.88)	91.92 (90.85–92.98)	12.12 (9.06–15.17)	11.81 (10.89–12.73)
**RT PCR status**								
Negative	89.37 (87.26–91.17)	87.75 (20.05–21.19)	8.64 (6.87–10.83)	9.35 (8.96–9.75)	87.91 (85.93–89.9)	86.91 (86.29–87.53)	8.7 (6.92–10.47)	9.26 (8.81–9.72)
Positive	94.77 (89.83–97.38)	93.29 (20.96–24.18)	17.64 (12.52–24.28)	14.42 (12.94–16.04)	93.26 (88.46–98.05)	93.56 (92.03–95.09)	20.89 (13.88–27.89)	18.82 (17.19–20.46)
**Vaccinated with at least one of the doses of approved vaccines**								
No	81.02 (78.26–83.51)	81.63 (17.95–18.66)	8.4 (6.85–10.26)	8.97 (8.60–9.35)	79.68 (77.17–82.2)	80.67 (80.14–81.19)	9.06 (7.29–10.82)	9.34 (8.89–9.79)
Yes	90.29 (88.56–91.78)	89.47 (21.03–21.97)	8.76 (7.48–10.24)	8.78 (8.55–9.02)	89.7 (88.18–91.22)	89.15 (88.78–89.52)	9.12 (7.78–10.47)	9.02 (8.7–9.33)

^∧^Adjusted for clustering.

**Table 5:** Total seropositivity among the participants.

	**Analysis of complete cases**		**Analysis of imputed data**	
	**Unweighted percent (95%CI)**	**Weighted percent (95%CI)**	**Unweighted percent (95%CI)**	**Weighted percent (95%CI)**
**Antibody type**
Seropositivity for Any of the three antibodies	86.18 (84.37–87.81)	85.49 (85.19–85.78)	85.21 (83.59–86.82)	84.84 (84.51–85.18)
Seropositivity for Anti Nucleotide IgG antibody	27.41 (25.06–29.88)	28.11 (27.622–28.61)	27.42 (25.23–29.6)	27.98 (27.48–28.49)^#^
Seropositivity for Anti Spike IgG antibody	85.52 (83.65–87.21)	84.84 (84.54–85.14)	84.45 (82.76–86.13)	84.08 (83.74–84.43)^##^
Seropositivity for Anti Spike IgM antibody	19.98 (18.53–21.51)	19.87 (19.58–20.16)	20.38 (18.96–21.8)	20.18 (19.84–20.52)^###^
Seropositivity for all three types of antibody	8.6(7.51–9.83)	8.87 (8.64–9.11)	9 (7.9–10.2)	9.18(8.91–9.45)

^#^Seropositivity adjusted for lower sensitivity and higher specificity = 27.88% (95% CI 27.38–28.39), for lower specificity and higher sensitivity = 27% (95% CI 26.52–27.53), and average sensitivity and specificity = 27.61% (95% CI 27.11–28.12).

^##^Seropositivity adjusted for lower sensitivity and higher specificity = 83.84% (95% CI 83.50–84.19), for lower specificity and higher sensitivity = 83.22% (95% CI 82.88–83.57), and average sensitivity and specificity = 83.63% (95% CI 83.29–83.98).

^###^Seropositivity adjusted for lower sensitivity and higher specificity = 19.92% (95% CI 19.58–20.26), for lower specificity and higher sensitivity = 19.42% (95% CI 19.08–19.76), and average sensitivity and specificity = 19.74% (95% CI 19.39–20.07).

### Attributable factors for seroprevalence in the general population

**Figure 4:**
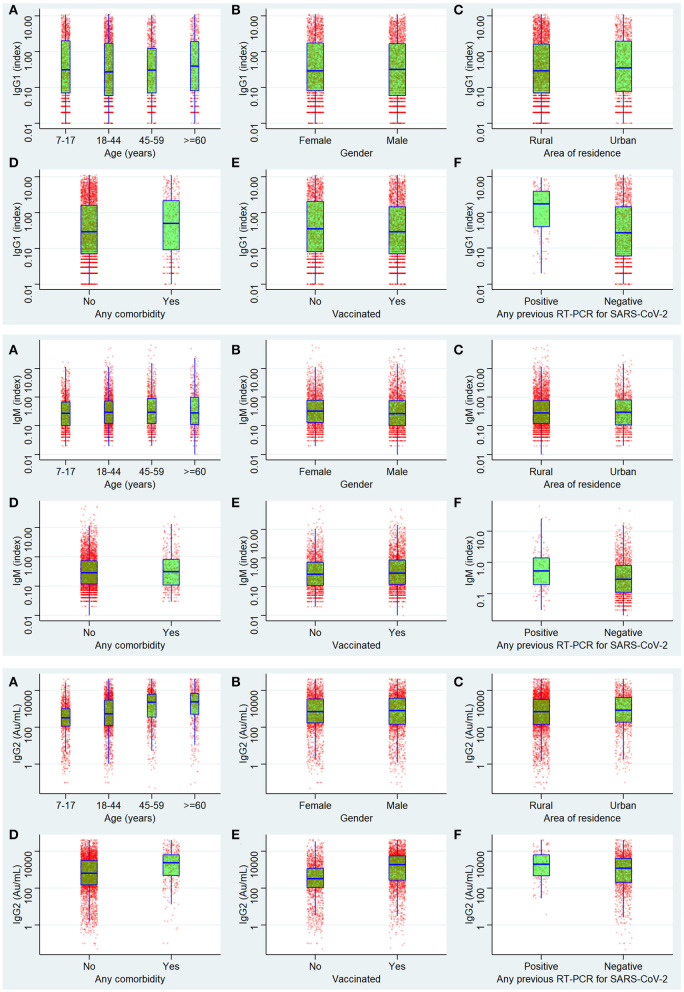
Antibody levels of IgG1 (anti-nucleocapsid antibody), IgM anti Spike antibodies, and IgG2 (anti-spike antibody).

The univariate logistic regression analysis showed that age, residence, comorbidity, vaccination, and results of the RT-PCR test were significantly attributed to affecting the seropositivity for any of the three antibodies. For anti-nucleocapsid IgG, the seropositivity was significantly affected by all variables except gender and age of more than 60 years. For the anti-spike IgG antibody, all the variables assessed were significantly affecting the seropositivity. For IgM, all were significant contributors except age <45 years and comorbidity. For the seropositivity for all three antibodies simultaneously, the variables significantly affecting the seroprevalence were all except the age group 45–59 years and vaccination status. In the multiple logistic regressions, the significant factors were the same as in the univariate analysis except for the age between 18 and 44 years regarding seropositivity for any of the three antibodies. For anti-nucleocapsid IgG, the significant predictors in multivariate logistic regression were age group 45–59 years, comorbidity, and vaccination status. For anti-spike IgG antibodies, the results did not change after multivariate analysis. The seropositivity of IgM was significantly associated with all the assessed variables in multiple regressions. Regarding the simultaneous seropositivity for all three antibody types, the significant predictors were all except age 44–59 years and vaccination status (Table 6) (Figure 4).

**Table 6:** Logistic regression analysis of the sample.

**Variables**	**Odds ratio for the Seropositivity of the antibodies (95%CI)**				
	**Any of the three antibodies**	**Anti N IgG antibodies**	**Anti Spike IgG antibody**	**Anti spike IgM antibody**	**All the three types of antibody**
**Univariate logistic regression**					
**Age group**					
18–44	1.33 (1.26–1.39)*	0.87 (0.82–0.91)*	1.31 (1.25–1.37)*	0.99 (0.92–1.04)	0.85 (0.78–0.92)*
45–59	2.45 (2.29–2.60)*	0.7 (0.66–0.73)*	2.15 (2.01–2.28)*	1.31 (1.24–1.37)*	0.99 (0.90–1.07)
≥60	2.71 (2.50–2.93)*	0.95 (0.90–1.00)	2.7 (2.48–2.92)*	1.55 (1.45–1.63)*	1.57 (1.45–1.70)*
**Gender**					
Female	1.27 (1.21–1.32)*	1.02 (0.98–1.06)	1.24 (1.19–1.29)*	1.19 (1.13–1.25)*	1.18 (1.10–1.25)*
**Residence**					
Rural	0.74 (0.69–0.79) *	0.89 (0.83–0.95)*	0.75 (0.70–0.79)*	0.88 (0.83–0.92)*	0.76 (0.71–0.82)*
**Comorbidity**					
Yes	2.16 (1.85–2.50) *	1.32 (1.21–1.43)*	2.21 (1.85–2.62)*	1.09 (0.98–1.19)	1.37 (1.23–1.52)*
**RTPCR test results**					
Positive	2.2 (1.66–2.88) *	3.05 (2.75–3.37)*	2.1 (1.63–2.70)*	1.37 (1.22–1.54)*	2.27 (2.01–2.55)*
**Vaccinated**					
Yes	1.97 (1.87–2.06) *	0.82 (0.79–0.85)*	1.97 (1.88–2.07)*	1.18 (1.12–1.24)*	0.96 (0.89–1.02)
**Multiple logistic regression**					
**Age group**					
18–44	0.94 (0.88–1.00)	0.94 (0.87–1.00)	0.91 (1.88–2.07)*	0.92 (0.86–0.97)*	0.89 (0.80–0.98)*
45–59	1.44 (1.32–1.57)*	0.73 (0.67–0.78)*	1.22 (3.80–4.05)*	1.2 (1.10–1.30)*	1.02 (0.90–1.14)
≥60	1.55 (1.38–1.74)*	0.95 (0.87–1.04)	1.48 (0.85–0.96)*	1.45 (1.32–1.57)*	1.61 (1.44–1.80)*
**Gender**					
Female	1.32 (1.25–1.37)*	0.98 (0.94–1.02)	1.29 (1.12–1.31)*	1.21 (1.15–1.27)*	1.16 (1.08–1.23)*
**Residence**					
Rural	0.73 (0.68–0.77)*	0.92 (0.86–0.98)*	0.73 (1.31–1.65)*	0.87 (0.82–0.92)*	0.78 (0.72–0.83)*
**Comorbidity**					
Yes	1.36 (1.15–1.61)*	1.47 (1.33–1.61)*	1.44 (1.23–1.34)*	0.86 (0.76–0.95)*	1.15 (1.01–1.30)*
**Vaccinated**					
Yes	1.84 (1.71–1.97)*	0.85 (0.79–0.90)*	1.92 (1.79–2.04)*	1.16 (1.08–1.24)*	0.93 (0.84–1.01)

^*^Denotes significant p-value for the relation (*p* < 0.05).

The seropositivity among healthcare workers was 89.24% (95% CI: 87.17 to 91.02%) for anti-spike IgG antibody; among police personnel, it was 95.5% (95% CI: 94.1 to 96.5%). Among the pregnant women, the seroprevalence for the same antibody was 74% (95% CI: 70 to 77·6%) (Supplementary Tables 3–5).

In Kashmir, the official statistics showed that a total of 195,152 cumulative cases were reported till 26 June 2021 and 125,102 between 23 December 2020 and 26 June 2021, with 85,992 total cases reported between 1 May and 26 June 2021. A total of 2,236 cumulative deaths were reported till 31 July 2021, with 1,036 deaths between 13 January 2021 and 31 July 2021 and 392 deaths from 1 May 2021 to 31 July 2021. The total infections based on seropositivity of anti-nucleocapsid IgG were 1,874,566 (95% CI: 1,826,972 to 1,922,160), and the infection to case ratio was 14.9 (95% CI: 14.6 to 15.36) from 23 December to 26 June 2021 and 14.7 (95% CI: 14.38 to 15.13) from 23 December to 5 July. Based on anti-spike IgG, the total cumulative infections were 5,029,091 (95% CI: 4,995,458 to 5,062,724), and the infection to case ratio was 25.77 (95% CI: 25.59 to 25.94) till 26 June 2021 and 25.5 (95% CI: 25.3 to 25.6) till 5 July 2021. Based on IgM, the cumulative infections were 1,167,003 (95% CI: 1,137,177 to 1,196,828), and the infection to case ratio was 13.5 (95% CI: 13.22 to 13.91) between 1 May and 26 June 2021 and 13.2 (95% CI: 12.94 to 13.61) from 1 May till 5 July 2021. In terms of all three antibody positivity, the cumulative number of infections was 592,703 (95% CI: 564,147 to 621,259), and the infection to case ratio was 6.89 (95% CI: 6.56 to 7.22) between 1 May and 26 June 2021; and 6.744 (95% CI: 6.41 to 7.06) between 1 May to 5 July. The death to infection ratio was 0.033% (95% CI: 0.034% to 0.032%) from 22 May to 31 July 2021 (3 weeks for the lag between infection to death) while calculating the seropositivity of IgM antibodies and 0.066% (95% CI: 0.0694 to 0.06309) with the proportion of seropositivity of all the three antibodies as the denominator. With IgG, the infection fatality rate was 0.055% (95% CI: 0.056% to 0.053%) between 13 January 2021 to 31 July 2021 using anti-nucleocapsid antibody seropositivity proportion and 0.04% (95% CI: 0.045 to 0.044%) using anti-spike antibody seropositivity proportion till 31 July 2021, respectively.

## Discussion

In this pan-Kashmir population-based cross-sectional study, we found that the adjusted seroprevalence of antibodies against SARS-CoV-2 (either IgG antibodies against Nucleocapsid or Spike antigen or IgM antibodies) was 84.84% (95% CI: 84.51 to 85.18) in the general population aged 7 years and more during the peak of the second wave of the pandemic in 2021. This increase in seroprevalence is unparallel though consistent with the increase in the number of COVID-19 cases reported between April and July 2021 in the districts surveyed. We observed high seropositivity (80.55%) among the participants aged younger than 18 years, despite the continued closure of schools and other educational institutes since the first wave and strict restrictions on social gatherings during the study period. Also, in this age group, the seropositivity for anti-nucleocapsid IgG antibodies was high compared to others, pointing toward the spread of infection to this group in recent months. The high number of cases and higher infectivity of the variant prevalent (15, 16) during the second wave led to the higher transmission among children and teens in the families even when schools had been closed for many months.

Our study describes one of the highest seroprevalences of COVID-19 in the world and is much higher than the recent study done among the Indian population by the Indian Council of Medical Research (ICMR) (9). Previously, high seroprevalence estimates in India were reported in Mumbai (17). In the year 2020, few other studies also reported high seroprevalence; in Brazil, researchers reported a seroprevalence of 44 to 66%, but most of the studies done in that year reported lower seroprevalence (18–21).

**Figure 5:**
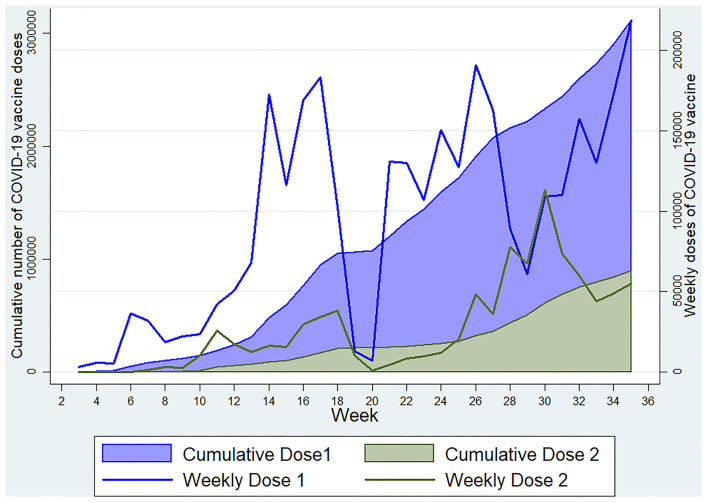
Weekly and cumulative doses of COVID-19 vaccine in Kashmir Division till 3 September 2021 (Data source: https://dashboard.cowin.gov.in).

We found that 27.98% of individuals were reactive to the antibodies against N-protein of SARS-CoV-2, possibly suggesting recent past infection or recent vaccination with a whole cell vaccine, but we found a higher seroprevalence of this antibody among those with no history of vaccination against COVID-19 with a significant association in the regression analysis. Also, more than 96% of those with a history of vaccination reported that vaccination was done with Covishield, which does not produce this antibody. Thus, the presence of anti-nucleocapsid antibody predominantly indicates a sign of recent infection, maybe within the past 3 to 4 months (22–26). Among the vaccinated ones having a history of vaccination with other than whole-cell vaccines, the presence of this antibody indicates pre-vaccination infection with the SARS-CoV-2. Similarly, the IgM assays showed that around one-fourth of the participants were positive, suggesting the presence of an acute type of antibody and, thus, a recent infection or recent vaccination. Our study showed a significantly higher prevalence of IgM antibodies in those who reported a positive history of vaccination as the vaccination drive was gaining maximum momentum around the study period. The seropositivity for IgG anti-spike antibody was also significantly higher among the participants who reported a positive history of vaccination, who were just more than half of the total participants. The Government of India initiated COVID-19 vaccination in January 2021, targeting healthcare and frontline workers in the first phase and individuals aged ≥60 years and those with chronic diseases in the age group of 45–59 years in the second phase (1), which later on was extended to include all individuals ≥18 years except lactating and pregnant women to whom vaccination was approved in July 2021. (27) During the initial months of vaccination drive, people were reluctant in Kashmir for vaccination. Since the wrath of the second wave, more people have started to accept the vaccination, and our data were collected during the peak of the second wave, after which vaccination started to gain more acceptance in Kashmir (Figure 5). As per official statistics, ~21.6 lakh people in the age group >18 years had received at least one dose of the COVID-19 vaccine till 16 July 2021.

Simultaneous positivity for all three antibodies was highest in participants more than 60 years. Looking at the statistics, it is revealed that approximately up to 13 to 17% of people aged more than 60 years were infected in the first wave and almost the same percentage in the second wave (28, 29), and this group was also among the first categories in which the vaccination was started and by the time of the second wave many of them were due for the second dose of their vaccine which can lead to seropositivity for anti-spike IgM and IgG antibodies. Furthermore, for the elderly who were positive previously or had their family members positive, it is expected to have more acceptive behavior and practice for the vaccine, so the presence of anti-nucleocapsid IgG antibody, as already stated, may be a sign of previous recent past infection in them (30–33). Those with comorbidity and with female gender also had a higher seroprevalence of the three antibodies as compared to their counterparts; same factors as stated in the elderly age can explain the scenario. Regarding females in India, studies have suggested higher mortality though fewer infections among them as compared to males (34), but across the globe, females have been found to experience less mortality (35), and in Kashmir, the resemblance was to the latter case. Fewer infections among them can be because of more asymptomatic or milder infections, thus not undergoing investigation and diagnosis. Likewise, our results showed acute antibodies, as well as anti-spike IgG, were simultaneously positive in them, even though females were reluctant to vaccination.

The urban/rural demarcation affected the seroprevalence, which was expected as the majority of COVID-19 cases in Kashmir were from urban areas. Some percentage of the participants with a positive test for coronavirus disease were also seronegative as research has shown differential antibody responses among the RT-PCR positive patients, especially for the asymptomatic patients having more possibility of being seronegative (36). Also, there can be a false negative antibody result owing to the limitation of the assays regarding their sensitivity and specificity. Similarly, the reason for those who had tested negative on RT-PCR being seropositive can be the false negative RT-PCR results or acquiring infection later on.

The infection fatality rate we found in our study ranges from 0.03 to 0.06%. Since IgM antibodies do not remain beyond 8 weeks after the infection and anti-nucleocapsid IgG antibody has also been seen to diminish within 3 months of the infection in various studies, so ~8 weeks and 3 months was used for the calculation pertaining to these antibodies regarding the infection to case ratio and infection fatality rate in the current study (37–40). Various systematic reviews and meta-analyses done on IFR have reported rates ranging from as low as 0.01 to 0.1% and as high as 5 to 10% in age-stratified results (41). While using anti-spike IgG seropositivity, these calculations were done with the total cumulative cases and cumulative deaths as the antibodies persist for years.

The countries with lower case fatality rates as compared to global rates had reported lower infection fatality rates, as low as reported in our study. These countries and areas include France, Brazil, Canada, several regions in China, Tokyo (Japan), and Karachi (Pakistan) (42–45). The reasons for lower mortality and lower infection fatality rates across different parts of the globe can be manifolds, including the social structure of the community, level of health care priority, age structure, overall development status of the community, and certain yet to be unfolded aspects like the effect of genetic makeup on the determinants of infections.

In the front line workers, we found the highest seroprevalence rates both in police as well as healthcare workers for anti-spike IgG antibodies but lower in the case of anti-nucleocapsid IgG and IgM antibodies. Both of these strata were exposed at a higher level than the general population to SARS-CoV-2 infections since the outbreak began, and both were also among the early recipients of vaccines.

The seropositivity among pregnant females was less than that found in the general population regarding anti-spike IgG but more in the case of anti-nucleocapsid IgG antibody, which points toward a level of infection in recent past in this strata, and furthermore, the pregnant females were least exposed to vaccination as it was not approved in them until July 2021.

Limitations: This study has a few limitations. First, the diagnostic accuracy of the antibody assays used in this study is variable. For the estimation of seroprevalence adjusted for sensitivity and specificity of the test, we relied on the accuracy estimates provided by the manufacturer. We did not estimate the diagnostic accuracy in-house. Second, seroconversion varies widely in persons with SARS-CoV-2 infection, with some people failing to mount a detectable humoral response while others show an overt response. Seroconversion might thus not provide an accurate picture of the infection.

In conclusion, the seroprevalence in Kashmir has reached exceptionally high levels during the second wave of the coronavirus pandemic. Owing to this extent of seropositivity, it is evident that a sizable number of unreported and undiagnosed cases have been there in the community during the second wave. Although the reported mortality rate has been on the lower side in Kashmir comparatively, the underreporting of positive cases might be a factor in the notable size of seropositivity in comparison to the number of reported cases. Pertinent to mention here, the vaccination could not be contributing much to this seroprevalence as the vaccination coverage was far less than this percentage during that period. Despite these results, the COVID-19 appropriate behavior and the vaccination guidelines need to be followed appropriately with continued improvement in its coverage, knowing the uncertainty of the immunity provided by the natural infection. Furthermore, researches need to be evoked to understand the effects of population dynamics on the COVID-19 pandemic.

## Data availability statement

The raw data supporting the conclusions of this article will be made available by the authors, without undue reservation.

## Ethics statement

The studies involving human participants were reviewed and approved by Institutional Ethics Committee, Government Medical College Srinagar. Written informed consent to participate in this study was provided by the participants/participants' legal guardian/next of kin.

## Author contributions

KL: data curation, validation, literature search, data analysis, data interpretation, writing–original draft, writing–review and editing, and project administration. SK: conceptualization, funding acquisition, writing–review and editing methodology, and resources. MQ, IH, SM, and MP: formal analysis, methodology, project administration, and writing–review and editing. JA, AhB, KB, AF, SB, MH, YJ, RRK, ZK, BM, FN, UQ, MWR, and MR: project administration, supervision, investigation, and writing–review and editing. AA, ArB, IC, SI, AJ, MFK, MAK, MSK, RK, AL, SN, TQ, RR, IS, and IAS: supervision, investigation, and writing–review and editing. All authors contributed to the article and approved the submitted version.

## Funding

This work was supported by the National Health Mission Jammu and Kashmir.

## Conflict of interest

The authors declare that the research was conducted in the absence of any commercial or financial relationships that could be construed as a potential conflict of interest.

## Publisher's note

All claims expressed in this article are solely those of the authors and do not necessarily represent those of their affiliated organizations, or those of the publisher, the editors and the reviewers. Any product that may be evaluated in this article, or claim that may be made by its manufacturer, is not guaranteed or endorsed by the publisher.
