# Supplementary material for: Seroprevalence of SARS-CoV-2-specific anti-spike IgM, IgG, and anti-nucleocapsid IgG antibodies during the second wave of the pandemic: A population-based cross-sectional survey across Kashmir, India
Source: Front Public Health. 2022 Oct 6;10:967447. doi: 10.3389/fpubh.2022.967447 (PMC9582950; doi:10.3389/fpubh.2022.967447)
Supplement: Supplementary file 1 [file Data_Sheet_1.docx]

**Table 1: District wise seroprevalence across Kashmir in the imputed data**

| **Un weighted analysis** | **Seropositivity for Any of the three types of antibody** | **Seropositivity for Anti N IgG antibody** | **Seropositivity for Anti Spike IgG antibody** | **Seropositivity for Anti Spike IgM antibody** | **Seropositivity for all the three types of antibody** |
| --- | --- | --- | --- | --- | --- |
| **Anantnag** | 87·2 (83·6-90·8) | 35·7 (29·2-42·3) | 86·9 (83·1-90·7) | 19·5 (15·7-23·2) | 10·6 (7·7-13·5) |
| **Bandipora** | 84·3 (78·5-90·2) | 26·2 (19·4-33·1) | 84·3 (78·5-90·2) | 22·8 (17·9-27·6) | 10·6 (6·4-14·9) |
| **Baramulla** | 85·4 (77·8-92·9) | 21·5 (14·7-28·4) | 84·4 (77-91·8) | 21·2 (16·6-25·9) | 8·7 (4·9-12·4) |
| **Budgam** | 84·8 (80·4-89·2) | 24·9 (19·5-30·2) | 84 (79·1-88·9) | 17·9 (15·2-20·7) | 7·6 (5·6-9·7) |
| **Ganderbal** | 82·4 (77·5-87·3) | 24·2 (20-28·5) | 81·7 (76·8-86·5) | 19·2 (16·6-21·8) | 5·7 (4·1-7·3) |
| **Kulgam** | 86·5 (82·9-90·1) | 27·2 (20·2-34·2) | 86 (82·3-89·8) | 15·9 (13·3-18·5) | 6 (3·8-8·2) |
| **Kupwara** | 84·2 (78·4-90) | 24 (19·4-28·7) | 82·9 (76·8-89) | 21 (16-26) | 7·5 (4·8-10·1) |
| **Pulwama** | 79·1 (75·2-83) | 28·2 (22·6-33·8) | 77·6 (73·1-82·1) | 23·7 (18·7-28·8) | 13·3 (8·3-18·3) |
| **Shopiyan** | 87·4 (83·8-90·9) | 26·9 (21-32·7) | 86·2 (82·1-90·2) | 17·6 (12·3-22·9) | 6·9 (3·8-10) |
| **Srinagar** | 89·9 (86·7-93·2) | 33·9 (25·3-42·5) | 89·5 (86·2-92·8) | 24·3 (20·5-28·1) | 13·1 (9·9-16·4) |
| **Weighted analysis** | | | | | |
| **Anantnag** | 87·5 (86·9-88·1) | 36·9 (36-37·9) | 87·5 (86·9-88·1) | 18·9 (18·3-19·6) | 9·7 (9·1-10·3) |
| **Bandipora** | 83·4 (82-84·8) | 25 (23·5-26·4) | 83·4 (82-84·8) | 21·7 (20·7-22·6) | 9·3 (8·6-10) |
| **Baramulla** | 84 (83·1-85) | 22 (21-22·9) | 83·1 (82·1-84·1) | 19·8 (18·9-20·8) | 8·4 (7·8-9) |
| **Budgam** | 83·4 (82·2-84·6) | 26·5 (24·6-28·3) | 82·8 (81·5-84·2) | 17·3 (15·6-18·9) | 8 (7-9) |
| **Ganderbal** | 82·2 (81·1-83·3) | 24·2 (23·3-25·2) | 81·5 (80·4-82·5) | 19·2 (18·6-19·9) | 5·6 (5·2-6) |
| **Kulgam** | 86·4 (85·6-87·2) | 29·2 (27·9-30·5) | 85·7 (84·9-86·5) | 15·5 (14·8-16·3) | 5·6 (5-6·1) |
| **Kupwara** | 83·9 (83·2-84·7) | 23·5 (22·8-24·2) | 82·4 (81·6-83·2) | 20 (19·3-20·8) | 6·7 (6·2-7·1) |
| **Pulwama** | 77·4 (76·4-78·3) | 27 (26·1-28) | 75·5 (74·7-76·4) | 23 (21·7-24·3) | 12·4 (11·4-13·4) |
| **Shopiyan** | 85·8 (84·9-86·7) | 28·2 (26·9-29·4) | 84·6 (83·7-85·5) | 17 (16-17·9) | 6·6 (6-7·1) |
| **Srinagar** | 88·6 (87·6-89·7) | 31·9 (29·7-34·2) | 88·1 (87·1-89·2) | 23·7 (22·5-24·8) | 12·7 (11·7-13·7) |

**Table 2: District wise seroprevalence across Kashmir in the complete case data and the available case data**

| **Districts** | **Seropositivity for Any of the three types of antibody** | **Seropositivity for Anti N IgG antibody** | **Seropositivity for Anti Spike IgG antibody** | **Seropositivity for Anti Spike IgM antibody** | **Seropositivity for all the three types of antibody** |
| --- | --- | --- | --- | --- | --- |
| **Results for data with no missed information of variables** | | | | | |
| **Anantnag** | 88·3 (84-91·6) | 36·2 (29·3-43·7) | 88·3 (84-91·6) | 19·1 (15·2-23·8) | 10·2 (7·4-14) |
| **Bandipora** | 84·5 (77·8-89·4) | 26·4 (20-33·9) | 84·5 (77·8-89·4) | 22·6 (17·9-28·2) | 10·7 (7-15·8) |
| **Baramulla** | 85·4 (75·9-91·5) | 21·6 (15·3-29·4) | 84·4 (75·3-90·5) | 20·8 (16·8-25·5) | 8·7 (5·6-13·4) |
| **Budgam** | 85·1 (79·8-89·1) | 24·7 (19·8-30·4) | 84·3 (78·3-88·8) | 17·9 (15·5-20·6) | 7·5 (5·7-9·9) |
| **Ganderbal** | 82·6 (77-87) | 24·3 (20·2-28·9) | 81·9 (76·4-86·3) | 19·2 (16·8-21·9) | 5·7 (4·3-7·6) |
| **Kulgam** | 87·7 (84·4-90·4) | 28·4 (21·5-36·5) | 87·2 (83·4-90·2) | 16·2 (13·6-19·2) | 6·1 (4-9) |
| **Kupwara** | 84 (77·1-89·1) | 23·6 (19·2-28·6) | 82·7 (75·5-88·1) | 21 (16·4-26·6) | 7·4 (5·1-10·6) |
| **Pulwama** | 82·1 (61·4-93) | 28·5 (18·7-40·9) | 82·1 (61·4-93) | 17·8 (5·3-45·4) | 7·1 (1·7-25·2) |
| **Shopiyan** | 87·4 (83·4-90·5) | 26·9 (21·4-33·2) | 86·2 (81·6-89·8) | 17·6 (12·9-23·6) | 6·9 (4·3-10·8) |
| **Srinagar** | 90·3 (86·3-93·2) | 34·1 (25·9-43·4) | 89·8 (85·8-92·8) | 24·4 (20·8-28·5) | 13·4 (10·4-17·1) |
| **Results for the data with the available information** | | | | | |
| **Anantnag** | 87·2 (83·2-90·4) | 35·8 (29·5-42·6) | 87 (82·7-90·3) | 19·4 (16-23·4) | 10·5 (8-13·8) |
| **Bandipora** | 84·3 (77·6-89·4) | 26·3 (20-33·7) | 84·3 (77·6-89·4) | 22·9 (18·4-28·1) | 10·7 (7·1-15·7) |
| **Baramulla** | 85·5 (76-91·6) | 21·5 (15·3-29·2) | 84·5 (75·4-90·6) | 21·2 (16·9-26·3) | 8·7 (5·6-13·3) |
| **Budgam** | 84·7 (79·5-88·7) | 24·5 (19·6-30) | 84 (78·1-88·4) | 17·7 (15·4-20·3) | 7·5 (5·7-9·7) |
| **Ganderbal** | 82·4 (76·9-86·8) | 24·2 (20·2-28·7) | 81·7 (76·3-86) | 19·2 (16·7-21·9) | 5·7 (4·3-7·6) |
| **Kulgam** | 86·4 (82·4-89·7) | 27·2 (20·7-34·8) | 86 (81·8-89·3) | 15·8 (13·4-18·6) | 5·9 (4-8·5) |
| **Kupwara** | 84·2 (77·5-89·1) | 24 (19·7-29) | 82·9 (75·9-88·2) | 21 (16·5-26·4) | 7·5 (5·2-10·6) |
| **Pulwama** | 79·2 (74·8-83) | 28·3 (22·8-34·6) | 77·7 (72·6-82·1) | 23·9 (19·1-29·4) | 13·5 (9·2-19·5) |
| **Shopiyan** | 87·4 (83·4-90·5) | 26·9 (21·5-33·2) | 86·2 (81·6-89·8) | 17·6 (12·9-23·6) | 6·9 (4·4-10·8) |
| **Srinagar** | 90·1 (86·3-93) | 34·1 (26-43·3) | 89·7 (85·8-92·6) | 24·5 (20·9-28·6) | 13·3 (10·4-16·9) |
| **Weighted results for the data having complete information of variables** | | | | | |
| **Anantnag** | 87·6 (87-88·1) | 37 (36·1-37·8) | 87·6 (87-88·1) | 18·9 (18·4-19·5) | 9·7 (9·2-10·2) |
| **Bandipora** | 83·5 (82·1-84·8) | 25·1 (23·7-26·5) | 83·5 (82·1-84·8) | 21·8 (20·8-22·7) | 9·4 (8·7-10·1) |
| **Baramulla** | 84·1 (83·3-84·9) | 22 (21·2-22·8) | 83·2 (82·4-84) | 19·7 (19·1-20·4) | 8·4 (8-8·9) |
| **Budgam** | 83·1 (82·5-83·8) | 26·1 (25·4-26·8) | 82·6 (82-83·3) | 16·9 (16·5-17·4) | 7·8 (7·5-8·1) |
| **Ganderbal** | 82·1 (81-83·2) | 24·3 (23·3-25·2) | 81·4 (80·3-82·5) | 19·2 (18·6-19·8) | 5·6 (5·2-6) |
| **Kulgam** | 86·3 (85·8-86·9) | 29·3 (28·1-30·5) | 85·7 (85·1-86·2) | 15·4 (14·8-15·9) | 5·4 (5-5·8) |
| **Kupwara** | 83·9 (83·2-84·7) | 23·5 (22·8-24·2) | 82·4 (81·6-83·2) | 20·1 (19·4-20·8) | 6·7 (6·3-7·1) |
| **Pulwama** | 75·8 (72·6-78·8) | 31·9 (29·4-34·4) | 75·8 (72·6-78·8) | 13·6 (11·4-16·1) | 5·3 (4·3-6·5) |
| **Shopiyan** | 85·7 (84·9-86·6) | 28·2 (27-29·4) | 84·6 (83·7-85·5) | 16·9 (16-17·9) | 6·6 (6-7·2) |
| **Srinagar** | 88·8 (87·8-89·6) | 32·2 (30·1-34·3) | 88·3 (87·3-89·2) | 23·9 (22·9-25) | 13 (12-13·9) |
| **Weighted results for the data with the available information** | | | | | |
| **Anantnag** | 87·6 (87-88·1) | 37 (36·1-37·9) | 87·6 (87-88·1) | 18·9 (18·3-19·4) | 9·7 (9·2-10·2) |
| **Bandipora** | 83·4 (82·1-84·7) | 25 (23·7-26·5) | 83·4 (82·1-84·7) | 21·7 (20·8-22·7) | 9·3 (8·7-10) |
| **Baramulla** | 84·1 (83·3-84·9) | 21·9 (21·1-22·8) | 83·2 (82·4-84) | 19·8 (19·1-20·4) | 8·4 (8-8·9) |
| **Budgam** | 83·2 (82·5-83·8) | 26·1 (25·4-26·8) | 82·7 (82-83·3) | 16·9 (16·5-17·4) | 7·8 (7·5-8·1) |
| **Ganderbal** | 82·2 (81-83·3) | 24·2 (23·3-25·2) | 81·4 (80·3-82·5) | 19·2 (18·6-19·9) | 5·6 (5·2-6) |
| **Kulgam** | 86·3 (85·8-86·8) | 29·3 (28·1-30·5) | 85·6 (85-86·2) | 15·4 (14·9-16) | 5·4 (5·1-5·8) |
| **Kupwara** | 83·9 (83·1-84·6) | 23·5 (22·8-24·2) | 82·3 (81·5-83·1) | 20 (19·3-20·8) | 6·7 (6·3-7·1) |
| **Pulwama** | 77·4 (76·7-78·2) | 27·3 (26·5-28·1) | 75·6 (74·9-76·3) | 23·2 (22·4-23·9) | 12·6 (11·9-13·3) |
| **Shopiyan** | 85·8 (84·9-86·6) | 28·2 (27-29·4) | 84·6 (83·7-85·5) | 17 (16-17·9) | 6·6 (6-7·2) |
| **Srinagar** | 88·8 (87·8-89·6) | 32·1 (30-34·3) | 88·3 (87·3-89·2) | 23·9 (22·8-24·9) | 12·9 (12-13·9) |

| **Base line characteristics (N=1024)** | **Number (percent)** | **Base line characteristics** | | **Number (percent)** |
| --- | --- | --- | --- | --- |
| **Gender** |  | **Symptoms in those positive for COVID-19** | | |
| Female | 342 (33·4) | Asymptomatic | | 64 (34·04) |
| Male | 682 (66·6) | Home treatment with oxygen | | 1 (0·53) |
| **Designation** | | Hospitalized | | 8 (4·26) |
| Administrative Staff | 105 (10·25) | Symptomatic, home treatment without oxygen | | 115 (61·17) |
| Ambulance Driver | 24 (2·34) | Total | | 188 (100) |
| Doctor | 284 (27·73) | **Whether any Family member was positive for COVID -19** | | |
| Field workers | 57 (5·57) | No | | 850 (83·01) |
| Paramedical | 519 (50·68) | Yes | | 174 (16·99) |
| Sanitation | 35 (3·42) | Total | | 1024 (100) |
| **Any Co morbidity** | | **Vaccinated** | | |
| No | 922 (90·04) | No | | 217 (21·19) |
| Yes | 102 (9·96) | Yes, 1 dose | | 200 (19·53) |
| **Type of co morbidity (frequency and percentage distribution out of total frequency of 123 among 102 participants )** | | Yes, 2 doses | | 607 (59·28) |
| Hypertension | 64 (52·03) | **Type of vaccine given** | | |
| Diabetes | 29 (23·57) | Covaxin | | 4 (0·5) |
| Thyroid disorder | 14 (11·38) | Covishield | | 802 (99·38) |
| COPD /asthma | 2 (1·62) | Don't | | 1 (0·12) |
| Any other | 14 (11·38) | **Reason if not vaccinated** | | |
| **Ever tested for COVID-19(RTPCR)** | | Do not believe | | 7 (3·23) |
| No | 313 (30·57) | Fear of vaccination | | 58 (26·73) |
| Yes | 711 (69·43) | Fear of vaccination ; not interested | | 1 (0·46) |
| **Results of the COVID-19 test** | | No response | | 32 (14·75) |
| Don't | 1 (0·14) | Not applicable | | 11 (5·07) |
| Negative | 522 (73·42) | Not interested | | 64 (29·49) |
| Positive | 188 (26·44) | Waiting foe due date | | 44 (20·28) |
| **Antibody seropositivity** | | | | |
| **Total seropositivity** | **Anti N IgG**  **(Total N=1004)** | **Anti Spike IgG**  **( Total N= 1004)** | **Anti Spike IgM**  **(Total N= 1004)** | **Seropositivity for Any of the three types of antibody (Total N=1004)** |
|  | 237 (23·61) | 896 (89·24) | 194 (19·32) | 928 ( 92·43) |
| **District wise Proportion of seropositivity** | | | | |
| **District** | **Anti N IgG**  **Percent (95%CI)** | **Anti Spike IgG**  **Percent (95%CI)** | **Anti Spike IgM**  **Percent (95%CI)** | **Seropositivity for Any of the three types of antibody** |
| Anantnag | 28·8 (20·7-38·6) | 90·7 (83·1-95·1) | 22·6 (15·4-32) | 95·9 (89·5 - 98·4) |
| Bandipora | 26·2 (18·5-35·8) | 82·8 (74-89) | 17·1 (10·9-25·9) | 82·8 (74·1 - 89·1) |
| Baramulla | 29·2 (21·1-38·9) | 93·9 (87·1-97·2) | 28·2 (20·2-37·9) | 93·9 (87·1 - 97·3) |
| Budgam | 17·6 (11·7-25·5) | 92·4 (86-96) | 15·9 (10·4-23·6) | 94·1 (88·2 - 97·2) |
| Ganderbal | 25·8 (18·7-34·5) | 91·3 (84·7-95·3) | 24·1 (17·2-32·7) | 92·2 (85·7 - 95·9) |
| Kulgam | 14·8 (9·2-22·8) | 87 (79·2-92·1) | 13·8 (8·5-21·7) | 91·7 (84·7 - 95·6) |
| Kupwara | 23·2 (15·9-32·5) | 88·8 (81-93·7) | 17·1 (10·9-25·9) | 88·9 (81·0 - 93·7) |
| Pulwama | 21·5 (12·3-34·9) | 96 (85·6-99) | 15·6 (8-28·3) | 96·1 (85·6 - 99·0) |
| Shopiyan | 27 (19·1-36·8) | 77 (67·6-84·4) | 29·1 (20·9-39) | 94·8 (88·1 - 97·8) |
| Srinagar | 22 (15·2-30·7) | 92·6 (86-96·2) | 8·26 (4·35-15·12) | 94·5 (88·3 - 97·5) |
| **Total Proportion of seropositivity** | **23·61(21·08-26·34)** | **89·24(87·17-91·02)** | **19·32(16·99-21·89)** | **92·4 (90·6 - 93·9)** |

**Table 3: Baseline characteristics and seroprevalence among health care workers across Kashmir**

**Table 4: Baseline characteristics and seroprevalence among police personnel across Kashmir**

| **Base line characteristics** | **Number (percent)** | | **Base line characteristics** | | **Number (percent)** |
| --- | --- | --- | --- | --- | --- |
| **Gender (N=1151)** | | | **Symptoms in those positive for COVID-19(N=93)** | | |
| **Female** | 7 (0·61) | | Asymptomatic | | 30 (32·26) |
| **Male** | 1144 (99·39) | | Symptomatic ;home treatment without oxygen | | 49 (52·69) |
| **Any Co morbidity (N=1151)** | | | Home treatment with oxygen | | 4 (4·3) |
| No | 1096 (95·22) | | Hospitalized | | 10 (10·75) |
| Yes | 55 (4·78) | | **Whether any Family member was positive for COVID-19 (N=1151)** | | |
| **Type of co morbidity (frequency and percentage distribution out of total frequency of 60 among 55 participants )** | | | **No** | | 1099 (95·48) |
| Hypertension | 21 (35) | | **Yes** | | 52 (4·52) |
| Diabetes mellitus | 13 (21·7) | | **Vaccinated (N=1151)** | | |
| Thyroid disorder | 9 (15) | | No | | 24 (2·09) |
| COPD/Asthma | 2 (3·3) | | Yes, 1 dose | | 108 (9·38) |
| Coronary heart disease | 4 (6·7) | | Yes, 2 doses | | 1019 (88·53) |
| Any other type | 11 (18·3) | | **Type of vaccine (N=1127)** | | |
| **Ever tested for COVID-19(RTPCR);(N=1151)** | | | Covishield | | 1123 (99·65) |
| No | 198 (17·2) | | Covaxin | | 3 (0·27) |
| Yes | 953 (82·8) | | Any other | | 1 (0·09) |
| **Results of the COVID-19 test(N=953)** | | | **Reason if not vaccinated (N=24)** | |  |
| Don't know /awaited | 2 (0·21) | | Do not believe | | 7 (29·17) |
| Negative | 858 (90·03) | | Fear of vaccination | | 9 (37·5) |
| Positive | 93 (9·76) | | No response | | 1 (4·17) |
|  |  | | Not interested | | 2 (8·33) |
|  |  | | Waiting for the due date | | 5 (20·83) |
| **Antibody seropositivity** | | | | | |
| **Total seropositivity** | **Anti N IgG(Total N=1123)** | **Anti Spike IgG( Total N= 1123)** | | **Anti Spike IgM(Total N= 1123)** | **Seropositivity for Any of the three types of antibody Total N=1123** |
|  | 209 (18·61) | 1072 (95·5) | | 146 (13) | 1,073 (95·5) |
| **District wise Proportion of seropositivity** | | | | | |
| **District** | **Anti N IgG**  **Percent (95%CI)** | **Anti Spike IgG**  **Percent (95%CI)** | | **Anti Spike IgM**  **Percent (95%CI)** | **Seropositivity for Any of the three types of antibody** |
| Anantnag | 14·7 (9·2 - 22·6) | 94·5 (88·3 - 97·5) | | 12·8 (7·8 - 20·5) | 94·5 (88·3 - 97·5) |
| Bandipora | 13·1 (7·8 - 21·3) | 93·9 (87·2 - 97·3) | | 12·1 (7·0 - 20·2) | 93·9 (87·2 - 97·3) |
| Baramulla | 16·0 (10·0 - 24·5) | 97·0 (91·1 - 99·0) | | 13·0 (7·7 - 21·1) | 97·0 (91·1 - 99·0) |
| Budgam | 22·2 (16·0 - 30·0) | 95·6 (90·5 - 98·0) | | 11·1 (6·8 - 17·6) | 95·6 (90·5 - 98·0) |
| Ganderbal | 22·0 (14·9 - 31·2) | 91·0 (83·6 - 95·3) | | 12·0 (6·9 - 20·0) | 91·0 (83·6 - 95·3) |
| Kulgam | 21·8 (14·8 - 30·9) | 96·0 (89·9 - 98·5) | | 14·9 (9·2 - 23·2) | 96·0 (89·9 - 98·5) |
| Kupwara | 19·0 (12·5 - 27·9) | 97·0 (91·1 - 99·0) | | 13·0 (7·7 - 21·1) | 97·0 (91·1 - 99·0) |
| Pulwama | 14·0 (9·5 - 20·0) | 94·8 (90·2 - 97·3) | | 15·7 (11·0 - 21·9) | 95·3 (91·0 - 97·7) |
| Shopiyan | 22·2 (15·4 - 31·0) | 98·1 (92·9 - 99·5) | | 10·2 (5·7 - 17·5) | 98·1 (92·9 - 99·5) |
| Srinagar | 23·7 (16·1 - 33·3) | 96·8 (90·5 - 99·0) | | 14·0 (8·3 - 22·6) | 96·8 (90·5 - 99·0) |
| **Total Proportion of seropositivity** | 18·6 (16·4 - 21·0) | 95·5 (94·1 - 96·5) | | 13·0 (11·2 - 15·1) | 95·5 (94·2 - 96·6) |

**Table 5: Baseline characteristics and seroprevalence among pregnant females across Kashmir**

| **Base line characteristics (N=484)** | **Number (percent)** | | **Base line characteristics** | **Number (percent)** |
| --- | --- | --- | --- | --- |
| **Any Co morbidity** | | | **Whether any Family member was positive for COVID -19** | |
| No | 453 (93·59) | | **No** | 467 (96·48) |
| Yes | 31 (6·404) | | **Yes** | 17 (3·512) |
| **Type of co morbidity (frequency and percentage distribution out of total frequency of 33 among 31 participants )** | | | **Vaccinated** | |
| COPD | 1 (3) | | No | 479 (98·96) |
| Diabetes | 8 (24·24) | | Yes, 1 dose | 4 (0·826) |
| Hypertension | 6 (18·18) | | Yes, 2 doses | 1(0·206) |
| Thyroid | 18 (54·55328) | |  |  |
| **Ever tested for COVID-19(RTPCR)** | | | **Type of vaccine given (N=5)** | |
| No | 156 (32·23) | | Covishield | 4(80) |
| Yes | 328 (67·76) | | Don't | 1 (20) |
| **Results of the COVID-19 test(N=328)** | | | **Reason if not vaccinated (N=479)** | |
| Don't know /awaited | 1 (0·3) | | Do not believe | 4(0·83) |
| Negative | 325 (99·09) | | Fear of vaccination | 11(2·296 |
| Positive | 2 (0·61) | | Fear of vaccination ; not applicable | 1 (·208) |
| **Symptoms in those positive for COVID-19** | | | No response | 47(9·81) |
| Asymptomatic | 1(50) | | Not applicable | 383(79·95) |
| Home treatment with oxygen | 1 (50) | | Not interested | 33(6·88) |
|  |  | | **If vaccine becomes available will you get vaccinated** | |
|  |  | | No | 222 (46·34) |
|  |  | | Yes | 257 (53·65) |
| **Antibody seropositivity** | | | | |
|  | **Anti N IgG**  **(Total N=423)** | **Anti Spike IgG (Total N=507)** | **Anti Spike IgM**  **(Total N= 469)** | **Seropositivity for Any of the three types of antibody (Total N=507)** |
| **Total seropositivity, n (%)** | 127 ( 30·0) | 375 (74) | 93 (19·8) | 384(75·7) |
| **District wise Proportion of seropositivity** | | | | |
| **District** | **Anti N IgG**  **Percent (95%CI)** | **Anti Spike IgG**  **Percent (95%CI)** | **Anti Spike IgM**  **Percent (95%CI)** | **Seropositivity for Any of the three types of antibody** |
| Anantnag | 32·0 (20·6 - 46·1) | 72·0 (58·1 - 82·7) | 14·0 (6·8 - 26·6) | 74·0 (60·2 - 84·3) |
| Bandipora | 20·4 (11·3 - 34·0) | 73·5 (59·5 - 83·9) | 14·3 (7·0 - 27·1) | 73·5 (59·5 - 83·9) |
| Baramulla | 38·0 (25·7 - 52·1) | 86·0 (73·4 - 93·2) | 24·0 (14·1 - 37·7) | 88·0 (75·7 - 94·5) |
| Budgam | 29·8 (19·4 - 42·9) | 78·9 (66·4 - 87·7) | 21·1 (12·3 - 33·6) | 80·7 (68·4 - 89·0) |
| Ganderbal | 16·0 (8·2 - 28·9) | 64·0 (49·9 - 76·0) | 18·0 (9·6 - 31·2) | 66·0 (51·9 - 77·7) |
| Kulgam^$^ | - | 40·7 (24·2 - 59·7) | - | 51·9 (33·6 - 69·7) |
| Kupwara | 28·6 (17·7 - 42·7) | 67·3 (53·1 - 79·0) | 18·4 (9·8 - 31·7) | 71·4 (57·3 - 82·3) |
| Pulwama | 38·0 (25·7 - 52·1) | 80·0 (66·6 - 88·9) | 20·0 (11·1 - 33·4) | 80·0 (66·6 - 88·9) |
| Shopiyan | 36·7 (24·5 - 51·0) | 81·6 (68·3 - 90·2) | 30·6 (19·3 - 44·8) | 81·6 (68·3 - 90·2) |
| Srinagar | 30·8 (12·0 - 59·2) | 70·0 (56·0 - 81·1) | 8·3 (1·2 - 41·5) | 70·0 (56·0 - 81·1) |
| **Total Proportion of seropositivity** | 30·0 (25·8 - 34·6) | 74·0 (70·0 - 77·6) | 19·8 (16·5 - 23·7) | 75·7 (71·8 - 79·3) |

^$ reagent was not available for anti N IgG and anti Spike IgM so samples could not be tested for these.^
